# Effects of Cylindrical Focused Ultrasound on Emulsifying Properties, Structural Characteristics, and Oxidative Stability of Liquid Egg Yolk

**DOI:** 10.1002/fsn3.72596

**Published:** 2026-11-13

**Authors:** Hyun Uk Cho, Chemin Nam, Prabhathma Yasasvi Rathnayake, So Eun Yeo, Seonae Hwangbo, Ki Ho Baek, Hae In Yong

**Affiliations:** ^1^ Department of Animal Science and Biotechnology Chungnam National University Daejeon Korea; ^2^ Animal Resources Food Tech Division National Institute of Animal Science, Rural Development Administration Wanju Korea; ^3^ R&D Center, Focused Ultra‐Sonic Tech. Lab. (FUST Lab) Daejeon Korea; ^4^ Advanced Bio and Healthcare Materials Research Division Korea Institute of Materials Science Changwon Korea

**Keywords:** cylindrical focused ultrasound, egg yolk, emulsifying functionality, emulsion stability, physicochemical properties

## Abstract

Cylindrical focused ultrasound enables controlled delivery of acoustic energy and has the potential to modify the functional properties of food materials. However, its effects on the emulsifying functionality of liquid egg yolk have not yet been investigated. Therefore, this study evaluated the effects of cylindrical focused ultrasound on the emulsifying properties, structural changes, quality characteristics, and oxidative stability of liquid egg yolk. Fresh egg yolk was treated with cylindrical focused ultrasound at 100 W under recirculating continuous‐flow conditions (20 mL/min) for 0 (control), 5, 10, and 20 min at 25°C. When liquid egg yolk was treated with cylindrical focused ultrasound for 20 min, emulsifying activity and emulsion stability increased from 0.03 to 0.10 and from 19.22% to 57.65%, respectively (*p* < 0.05). The creaming index was significantly lower with cylindrical focused ultrasound treatment for 20 min (28.26%) than in the control (43.18%) at the end of the 60 min monitoring period (*p* < 0.05). As the cylindrical focused ultrasound treatment time increased to 20 min, particle size decreased, while surface hydrophobicity and pH increased (*p* < 0.05). Cooking yield and CIE a* and b* values of liquid egg yolk were not significantly affected by cylindrical focused ultrasound treatment (*p* > 0.05). Meanwhile, the TBARs value significantly increased from 0.767 to 1.196 mg MDA/L after 20 min of treatment (*p* < 0.05), indicating increased lipid oxidation. These findings suggest that cylindrical focused ultrasound has potential for improving the emulsifying functionality of liquid egg yolk, accompanied by changes in structural characteristics. Although increased lipid oxidation presents a limitation, further optimization may enable the application of this technology to emulsion‐based food systems.

## Graphical abstract

Cylindrical focused ultrasound was applied as a continuous‐flow, non‐thermal processing technology (100 W, 20 mL/min, 25°C) to modify the functional and physicochemical properties of liquid egg yolk. The acoustic cavitation generated by cylindrical focused ultrasound induced structural changes in egg yolk proteins, reducing particle size and increasing surface hydrophobicity. Consequently, cylindrical focused ultrasound treatment significantly enhanced the emulsifying activity, emulsion stability, and creaming stability of liquid egg yolk without compromising cooking yield, demonstrating its potential for industrial applications in emulsion‐based food systems.

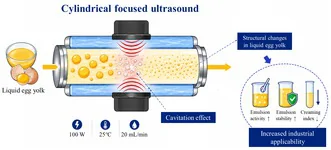

## 1 Introduction

Egg yolk is widely used as a natural emulsifier because of its complex composition of proteins, phospholipids, and lipoproteins. Proteins contribute to interfacial adsorption and film formation through structural rearrangement, whereas amphiphilic phospholipids facilitate stabilization at the oil–water interface (Jung et al. 2025). Egg yolk consists mainly of plasma and granules, which differ in their emulsifying roles (Tian et al. 2024). Low‐density lipoproteins (LDL), abundant in the plasma fraction, readily adsorb at the oil–water interface and contribute to emulsion stability (Mine 1998; Le Denmat et al. 2000). In contrast, high‐density lipoproteins (HDL) are mainly associated with granules together with phosvitin, and their emulsifying behavior depends on the aggregation and dispersion state of the granules (Le Denmat et al. 2000). These properties enable egg yolk to be widely used in emulsion‐based foods such as mayonnaise, dressings, sauces, and bakery products (Nam et al. 2026).

Therefore, the emulsifying performance of egg yolk is strongly influenced by the structural organization and interfacial behavior of its components. Protein conformation, lipoprotein aggregation, particle organization, and exposure of hydrophobic regions can affect adsorption at the oil–water interface and subsequent emulsion stability (Ribeiro et al. 2023). Previous studies have shown that physical, chemical, and biological modifications of egg proteins can alter their emulsifying properties by modifying protein structure and interfacial behavior (Tian et al. 2024). Although egg yolk is known to have excellent emulsifying ability, this functionality can be reduced during processing involving heat or prolonged storage, mainly due to excessive aggregation of egg yolk components (Hatta et al. 2018; Wang et al. 2024). In contrast, moderate structural modifications under appropriate processing conditions may improve emulsifying properties by facilitating the dispersion of particles and the formation of yolk‐based emulsions (Rathnayake, Yu, et al. 2025).

Recently, non‐thermal processing technologies have attracted increasing attention for improving the functional properties of foods (Cho et al. 2026). In egg yolk, various non‐thermal processing approaches have been investigated to improve emulsifying properties and related physicochemical characteristics (Suhag 2024). Among these non‐thermal processing technologies, ultrasound has been widely used in liquid food systems for its ability to enhance dispersion and emulsification (Yoshimasa et al. 2024). Previous studies reported that ultrasound treatment can induce moderate structural modifications in food components, thereby improving emulsifying properties and dispersion stability (Patil et al. 2022; Xie et al. 2020). However, most previous studies used horn‐ or bath‐type ultrasound systems, which can lead to localized energy delivery, inconsistent cavitation distribution, and limited uniformity of processing in liquid foods (Moholkar et al. 2000). In particular, these conventional systems may have limitations in continuous processing and precise control of acoustic energy during liquid food treatment (Yun et al. 2023).

To address these limitations, cylindrical focused ultrasound systems have been developed with a cylindrical piezoelectric configuration designed to direct acoustic energy circumferentially toward the central region (Hwangbo et al. 2021; Yu et al. 2026; Yun et al. 2023). Yun et al. (2023) reported that focused ultrasound produced smaller nanoliposomes with a narrower particle size distribution and improved dispersion stability compared with conventional horn‐ and bath‐type ultrasound systems. These findings suggest that the focused acoustic configuration may facilitate particle disruption and dispersion, highlighting its potential for modifying particle organization and interfacial properties in liquid food systems. Therefore, we hypothesized that the application of cylindrical focused ultrasound to liquid egg yolk would induce cavitation effects throughout the treatment region, leading to structural modifications of egg yolk components and consequently affecting their emulsifying properties. Accordingly, this study investigated the effects of cylindrical focused ultrasound treatment time on the emulsifying activity, emulsion stability, and creaming index of liquid egg yolk, together with associated changes in particle size, surface hydrophobicity, quality characteristics, and oxidative stability.

## 2 Materials and Methods

### 2.1 Preparation of Egg Yolk Samples

Hy‐line brown (58‐week, caged, on standard diets) eggs laid within 24 h were obtained from the Animal Research Center of Chungnam National University (Chenongyang, Korea) and were used in the study. Egg yolks from 30 eggs were pooled and thoroughly mixed before treatment to ensure a uniform initial sample composition. The mixed egg yolk was divided into 50 mL aliquots, and three separate aliquots were independently treated for each treatment time (0, 5, 10, and 20 min). All treatment samples were prepared from the same pooled egg‐yolk batch.

### 2.2 Application of Cylindrical Focused Ultrasound Treatment

As indicated in Figure 1, sonication was treated using a focused ultrasound system (DEBREX; FUST Lab Co. Ltd., Daejeon, Republic of Korea) equipped with a single‐lead zirconate titanate (PZT) transducer. The cylindrical focused ultrasound device was operated at a power output of 100 W for all runs (Kim et al. 2025; Rathnayake et al. 2026). A 50 mL egg yolk sample was continuously recirculated through the device at a flow rate of 20 mL/min for total treatment times of 5, 10, or 20 min, while the untreated sample (0 min) served as the control. The temperature of the egg yolk was maintained and monitored at room temperature (25°C) during cylindrical focused ultrasound treatment using a circulating water bath. After cylindrical focused ultrasound treatment, the egg yolk samples were directly cooled and maintained at 4°C until further analysis.

**FIGURE 1:**
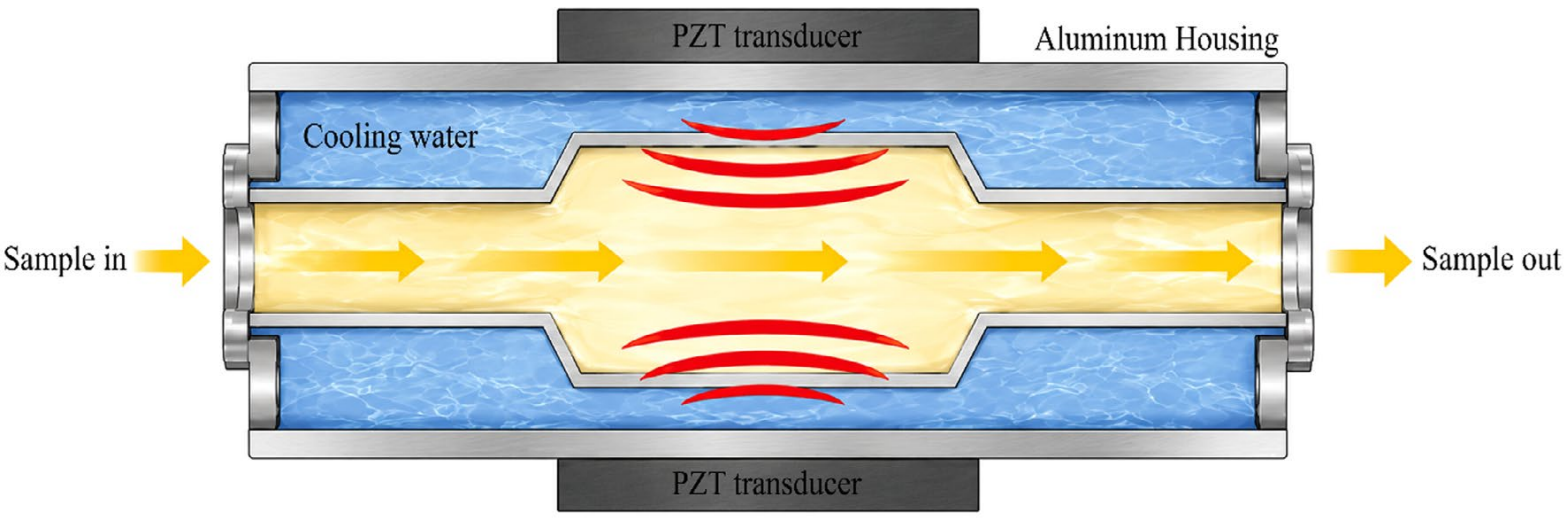
Schematic diagram of the single zirconate titanate (PZT) transducer for the preparation of cylindrical focused ultrasound.

The treatment times were selected based on preliminary experiments with liquid egg yolk and our previous study using the same cylindrical focused ultrasound system. Our previous study demonstrated that cylindrical focused ultrasound treatment could improve the functional properties of liquid egg white while largely maintaining protein integrity (Rathnayake et al. 2026). Accordingly, treatment times of 5, 10, and 20 min were selected to evaluate time‐dependent changes in the physicochemical and functional properties of liquid egg yolk.

### 2.3 Determination of Physicochemical Effect by Cylindrical Focused Ultrasound Treatment

#### 2.3.1 Iodide Oxidation Method

The generation of hydroxyl (•OH) radicals during ultrasonic treatment was indirectly evaluated using the iodide oxidation method in an aqueous KI system under the same ultrasonication conditions used for the egg‐yolk experiments. First, the potassium iodide (KI) solution was prepared using ultrapure water produced by a water purification system (Dream Mega, PULEUNLAB, Osan, Republic of Korea), with a resistivity of 18.2 MΩ·cm. Then, the KI solution (1 g/L, 50 mL) was exposed to ultrasound treatment for 0, 5, 10, and 20 min. In this study, the absorbance of triiodide ions (I_3_^−^) was measured at 351 nm using a UV–Vis spectrophotometer (HACH DR3900, Hach Company, Loveland, United States).

#### 2.3.2 Calculation of Delivered Sonic Energy (DSE)

Ultrasonication dosage was quantified as delivered sonic energy (DSE, kJ/mL), which represents the actual ultrasonic energy delivered to the sample. Delivered sonic energy (DSE) was calculated as described in our previous study (Rathnayake, Kim, et al. 2025). The calculated DSE values were used to compare ultrasonic energy input under different treatment times. Based on treatment times of 5, 10, and 20 min with a 50 mL sample, the calculated DSE values were 13.2, 26.5, and 53.0 kJ/mL, respectively (Data not shown).

### 2.4 Evaluation of Emulsifying Properties

#### 2.4.1 Emulsifying Activity and Emulsion Stability

The emulsifying activity of egg yolk was evaluated using a turbidimetric method, as previously described by Li et al. (2022), with slight modifications. To prepare the egg yolk emulsion, 30 mL of 0.5% (w/v) egg yolk solution was mixed with 40 mL of soybean oil and homogenized at 8000 rpm for 1 min using a homogenizer (T10 Basic, Ika Co., Staufen, Germany). Subsequently, 10 μL of the egg yolk emulsion was collected from the bottom of the beaker at 0 and 10 min after homogenization and added to 30 mL of 0.1% (w/v) SDS solution, respectively. The absorbance of the diluted egg yolk emulsions was measured at 500 nm using a UV–VIS spectrophotometer (Ubi 490; MicroDigital Co. Ltd., Seoul, Republic of Korea), and 0.1% SDS solution was used as a blank. Emulsifying activity (A_0_) and emulsion stability were calculated according to the following equations:

$$\text{Emulsifying activity}=A_{0}$$

$$\text{Emulsion stability}=\left(A_{0}\times ∆T\right)/\left(A_{0}-A_{10}\right)$$

where A_0_ is the absorbance at 0 min, and A_10_ is the absorbance at 10 min, ΔT means the time interval.

#### 2.4.2 Creaming Stability

The creaming stability of egg yolk emulsions was evaluated using the method described by Yu et al. (2022), with slight modifications. Briefly, 0.6 mL of egg yolk was diluted with 10 mL of distilled water. Then 10 mL of soybean oil was added to the mixture. The mixture was homogenized at 10,000 rpm for 40 s to obtain an emulsion. The egg yolk emulsion was transferred into a glass graduated cylinder and left to stand at room temperature for 1 h. The creaming index (%) was determined by measuring the height of the clear serum layer at 5 min intervals. The creaming index was calculated according to the following equations:

$$\text{Creaming index}\;\left(\%\right)=H_{s}/H_{t}\times 100$$

where H_s_ is the height of the clear sedimented phase (cm) and Ht (cm) is the height of the total emulsion (cm).

### 2.5 Particle Size Distribution

The particle size distribution of egg yolk was measured following Rathnayake, Kim, et al. (2025) and Rathnayake, Yu, et al. (2025) using a particle analyzer (Litesizer 500; Anton Paar GmbH, Graz, Austria). Egg yolk samples were diluted in ultrapure water at a ratio of 1:1000 (v/v). Then, a 1.2 mL sample of the diluted egg yolk was transferred to a cuvette, and the particle size was determined.

### 2.6 Optical Microscope Observation

The egg yolk emulsion was prepared as described previously. Then, microscopic observation was performed according to the method described by Rathnayake et al. (2026). The egg yolk emulsion was placed on a glass slide, covered with a coverslip, and observed using an Olympus BX‐53 microscope (Olympus Optical Co. Ltd., Tokyo, Japan). After equilibrating the samples for 2 min at room temperature, micrographs were captured at 20X magnification.

### 2.7 Surface Hydrophobicity

The surface hydrophobicity of egg yolk following cylindrical focused ultrasound treatment was analyzed by the fluorescence detection of 1‐(anilino)naphthalene‐8‐sulfonate (ANS, A1028, Sigma‐Aldrich, St. Louis, MO, USA). Each sample was prepared at five different concentrations (0.1, 0.2, 0.3, 0.4, and 0.5 mg/mL). Then, 1.8 mL of the sample solution was mixed with 18 μL of 8 mM ANS dissolved in 50% (v/v) methanol and incubated in the dark for 20 min. The fluorescence intensity was then measured using a multimode microplate reader (Varioskan LUX; Thermo Fisher Scientific, Waltham, MA, USA) at excitation and emission wavelengths of 370 and 470 nm, respectively. Surface hydrophobicity was expressed as the slope of the linear regression of fluorescence intensity on protein concentration, which was used as an indicator of hydrophobicity.

### 2.8 pH

The pH of egg yolk samples was measured at room temperature using a calibrated pH meter (PM‐3; CAS Co. Ltd., Seoul, Republic of Korea). Before measurement, each sample was gently mixed using a vortex mixer to ensure uniformity. The instrument was standardized with buffer solutions of pH 4.01, 7.00, and 10.00 before use to obtain accurate readings.

### 2.9 Thiobarbituric Acid Reactive Substances (TBARs)

Lipid oxidation in egg yolk samples was determined by quantifying the malondialdehyde (MDA) content using the thiobarbituric acid reactive substances (TBARs) assay as described by Rathnayake, Kim, et al. (2025) and Rathnayake, Yu, et al. (2025) with slight modifications. A thiobarbituric acid (TBA)/trichloroacetic acid (TCA) reagent containing 20 mM TBA and 15% (w/v) TCA was prepared in NaOH solution. Briefly, 1 mL of egg yolk sample was mixed with 20 μL of 7.2% (w/v) butylated hydroxytoluene in a 15 mL conical tube and vortexed. Subsequently, 2 mL of TBA/TCA reagent was added, and the mixture was vortexed again. The mixture was then incubated in a water bath at 90°C for 30 min and cooled to room temperature. After cooling, the samples were centrifuged at 3500 rpm for 15 min at 4°C. The absorbance of the supernatant (200 μL) was measured at 532 nm using a multimode microplate reader. The concentration of MDA was quantified from a standard curve constructed with 1,1,3,3‐tetraethoxypropane, and the results were expressed as milligrams of MDA per liter (mg MDA/L).

### 2.10 Color Values

The color characteristics of egg yolk samples were measured using a colorimeter (CM‐5; Konica Minolta, Osaka, Japan) based on the International Commission on Illumination (CIE) system. The parameters CIE L* (lightness), CIE a* (redness/greenness), and CIE b* (yellowness/blueness) were recorded. The instrument was calibrated with a standard white plate and distilled water before measurement. The total color difference (ΔE) was calculated using the following equation:

$$∆E=\sqrt{{\left(∆\mathrm{CIE}\;L^{*}\right)}^{2}+{\left(∆\mathrm{CIE}\;a^{*}\right)}^{2}+{\left(∆\mathrm{CIE}\;b^{*}\right)}^{2}}$$

### 2.11 Cooking Yield

About 50 g of egg yolk was placed on a stainless dish and heated in a water bath (HB‐205SW; Hanbeak Scientific Technology, Gyeonggi‐do, Republic of Korea) at 90°C for 30 min. The masses of raw and cooked egg yolk were measured to determine the cooking yield. The samples before and after heating were weighed as m_1_ and m_2_, respectively. The cooking yield (%) was calculated according to the following equation:

$$\text{Cooking yield}\;\left(\%\right)=\frac{m_{2}}{m_{1}}\times 100$$

where m_1_ represents the mass (g) of the sample before cooking, and m_2_ represents the mass (g) after cooking.

### 2.12 Statistical Analysis

For each treatment condition, three separate aliquots prepared from the pooled egg‐yolk batch were independently subjected to the respective treatment and were considered independent experimental replicates. Thus, a total of 12 independently treated samples were obtained across the four treatment conditions (three replicates × four treatments). The results were statistically analyzed using SPSS software (version 29; IBM Corp., Armonk, NY, USA). Data normality was assessed using Shapiro–Wilk, and homogeneity of variances was evaluated using Levene's test. Then, one‐way analysis of variance (ANOVA) was performed to determine significant differences among treatments, followed by Tukey's multiple comparisons test at a significance level of *p* < 0.05.

## 3 Results and Discussion

### 3.1 Emulsifying Properties

#### 3.1.1 Emulsifying Activity and Emulsion Stability

The emulsifying activity (A_0_) of egg yolk without cylindrical focused ultrasound treatment was 0.035 (Figure 2A). After cylindrical focused ultrasound treatment for 5, 10, and 20 min, A_0_ increased significantly compared with the A_0_ of untreated egg yolk (*p < 0.05*). In particular, the A_0_ of the egg yolk treated with cylindrical focused ultrasound for 20 min was highest (0.102), which was approximately threefold higher than that of the untreated egg yolk. Emulsifying activity is the ability of egg yolk components to rapidly adsorb at an oil–water interface and form an initial interfacial film (Yang et al. 2025). Therefore, the increased emulsifying activity observed after cylindrical focused ultrasound treatment suggests improved participation of surface‐active egg yolk components in emulsion formation.

**FIGURE 2:**
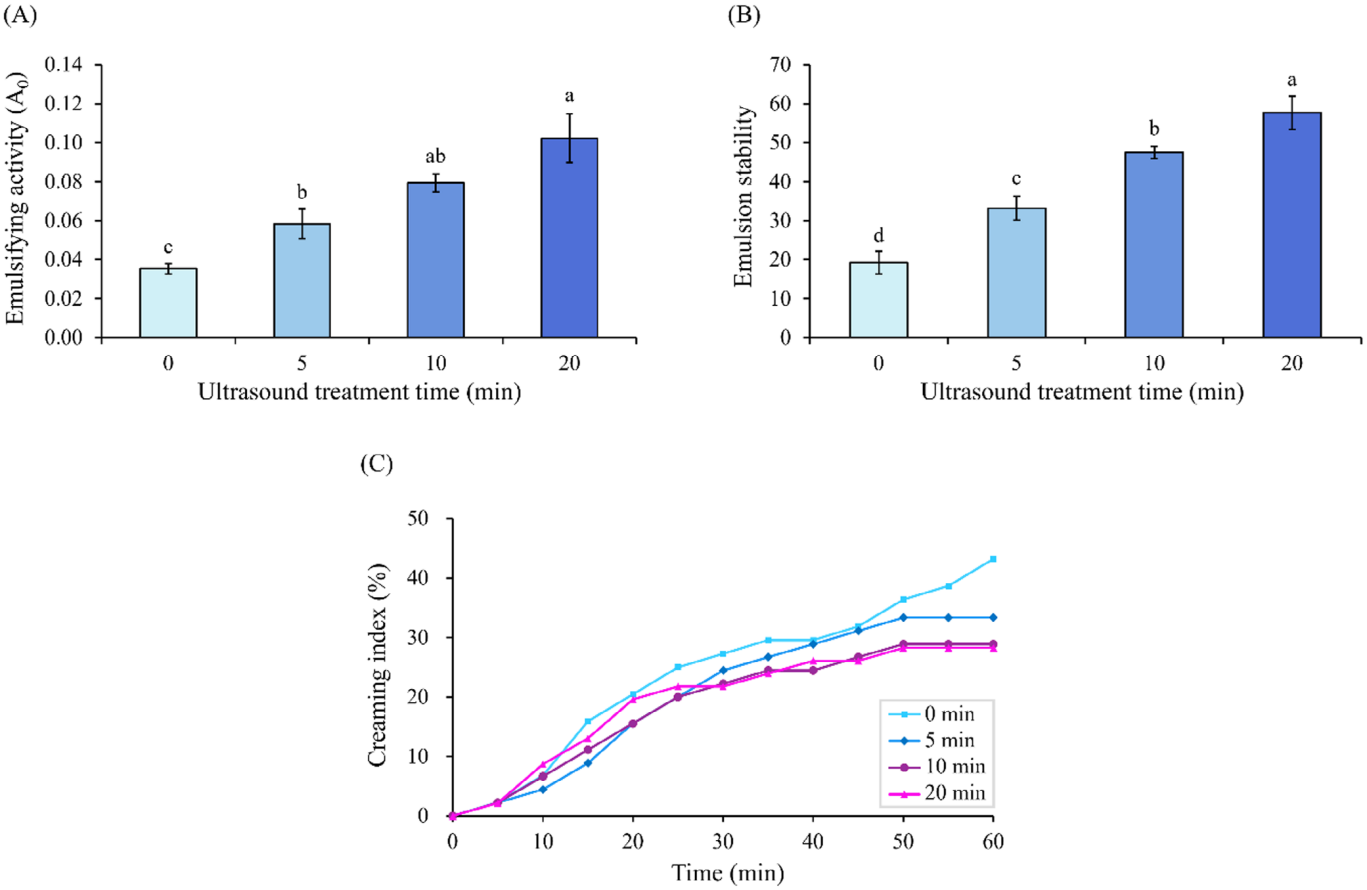
Effect of cylindrical focused ultrasound treatment time on the emulsifying properties of egg yolk: (A) Emulsifying activity, (B) Emulsion stability, and (C) Creaming index. ^a–d^Values with different superscripts differ significantly (*p* < 0.05) among treatments.

Similar to emulsifying activity results, the emulsion stability of egg yolk significantly increased with longer cylindrical focused ultrasound treatment time (*p* < 0.05; Figure 2B). Emulsion stability refers to the resistance of a preformed emulsion to destabilization, such as creaming, flocculation, and coalescence, during storage (Hodge and Rousseau 2005). Consistent with this study, Xie et al. (2020) observed that ultrasound significantly increased emulsifying activity and emulsion stability of liquid egg yolk. However, depending on processing intensity and matrix conditions, ultrasound may also cause particle flocculation or recoalescence, which can adversely affect interfacial properties and emulsion stability (Li et al. 2022; Scudino et al. 2020). However, within the treatment range examined in the present study (up to 20 min), cylindrical focused ultrasound did not induce recoalescence‐related deterioration, whereas emulsifying activity and emulsion stability of egg yolk increased within the treatment range examined in this study.

#### 3.1.2 Creaming Stability

The creaming index reflects the gravitational separation of oil droplets in an emulsion system, with lower values indicating higher creaming stability (McClements 2004). To analyze the creaming stability of egg yolk following cylindrical focused ultrasound treatment time, the creaming index of egg yolk was monitored for 60 min. As shown in ultrasound treatment time, the creaming Figure 2C the creaming index of egg yolk progressively increased over time for all groups, indicating gradual phase separation. However, the creaming index of egg yolk was influenced by cylindrical focused ultrasound treatment time. At the end of the 60 min monitoring, the untreated egg yolk indicated the highest creaming index (43.18%), whereas the 5, 10, and 20 min cylindrical focused ultrasound‐treated egg yolk emulsions showed substantially lower values of 33.33%, 28.89%, and 28.26%, respectively.

The lower creaming index of the cylindrical focused ultrasound‐treated samples indicates improved resistance to gravitational separation. This trend was consistent with the increased emulsifying activity and emulsion stability shown in Figure 2A,B. Creaming is one of the most common forms of physical instability in emulsions, and emulsions with larger or more polydisperse droplets generally show faster phase separation because gravitational separation becomes more pronounced (McClements 2004; Dickinson 2013). Therefore, the reduced creaming index observed after cylindrical focused ultrasound treatment provides further evidence that the physical stability of egg yolk emulsions was improved.

Similar improvements in egg yolk creaming index after ultrasound treatment have been reported previously. Yu et al. (2022) applied high‐intensity ultrasound at 40 kHz with powers of 240 and 480 W for 10, 20, and 30 min to enzymatically hydrolyzed egg yolk. They found that ultrasound‐mediated dissociation of yolk granules resulted in smaller emulsion droplets and reduced creaming rates. In addition, Yoshimasa et al. (2024) reported that high‐intensity ultrasound treatment of diluted liquid egg yolk at 210 W for 9 min resulted in the highest creaming stability index. The continuous decrease in creaming index with increasing cylindrical focused ultrasound time in the present study further supports the role of ultrasound in producing emulsions with improved creaming stability.

### 3.2 Particle Size Distribution of Egg Yolk and Microstructure of the Egg Yolk Emulsion

To verify whether the improvements in emulsifying activity, emulsion stability, and creaming stability were associated with changes in the physical structure of the system, the particle size of egg yolk was analyzed (Figure 3A). Compared with the untreated egg yolk, the curves for the egg yolk treated with cylindrical focused ultrasound for 5 and 10 min shifted leftward, indicating smaller particles. Moreover, the egg yolk treated for 20 min resulted in the smallest particle size distribution among all treatments and shifted most to the left in the distribution curve. These results showed that cylindrical focused ultrasound progressively reduced the particle size of egg yolk as the treatment time increased.

**FIGURE 3:**
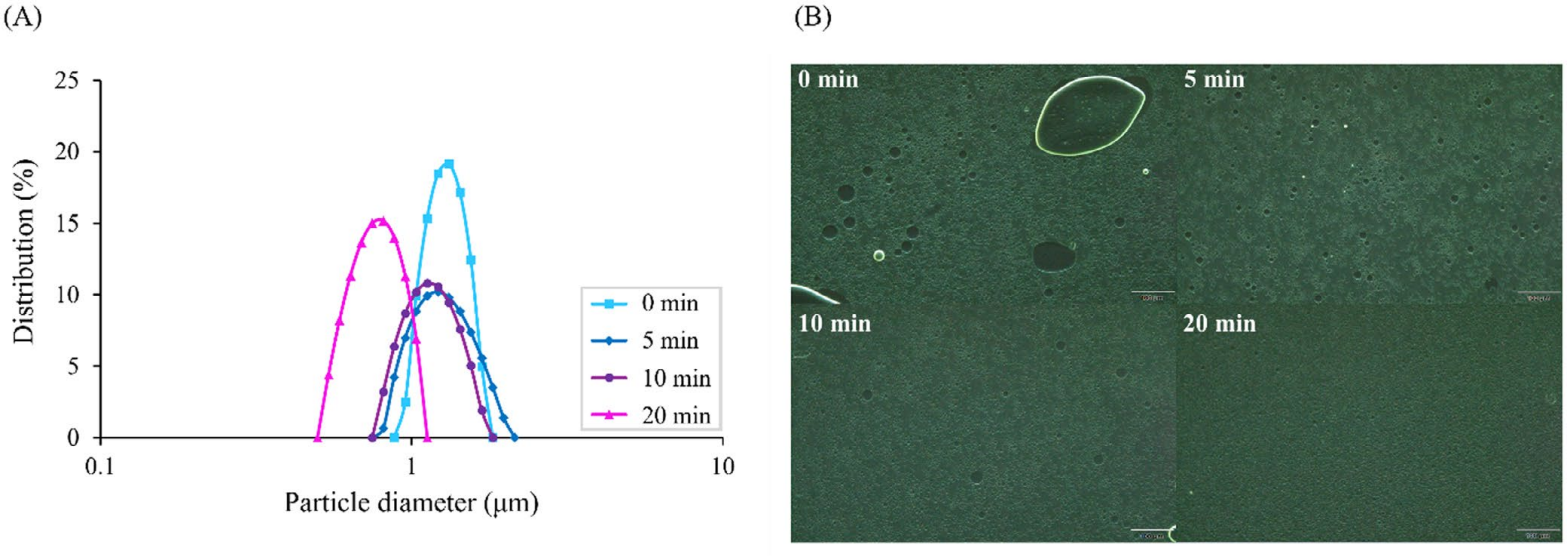
Effect of cylindrical focused ultrasound treatment time on particle size distribution of egg yolk and microstructure of egg yolk emulsions: (A) Particle size distribution curves of egg yolk. (B) Optical microscopy images of egg yolk emulsions.

Cylindrical focused ultrasound can generate both physical and chemical effects in liquid (Rathnayake, Kim, et al. 2025). The reduction in particle size was mainly in relation to the physical effect of cylindrical focused ultrasound. In this study, DSE was used as an index of the acoustic energy actually transferred to the egg yolk during cylindrical focused ultrasound. The calculated DSE values increased with treatment time, reaching 13.2, 26.5, and 53.0 kJ/mL for 5, 10, and 20 min, respectively (Data not shown). This result indicates that longer treatment delivered greater physical acoustic energy to the egg yolk system.

When the acoustic intensity of ultrasound exceeds a threshold, cavities (gas or bubbles) are formed in the liquid (Rathnayake, Yu, et al. 2025). These bubbles grow and subsequently collapse violently, known as acoustic cavitation. Cavitation collapse generates high temperatures and pressures with physical effects such as shock waves, microjets, intense shear forces, and turbulence in the surrounding liquid (Yu et al. 2026). During cylindrical focused ultrasound treatment, cavitation‐induced shear forces and turbulence may disrupt large yolk aggregates and improve their dispersion, which is consistent with the progressive reduction in particle size observed in the present study. A reduction in particle size can increase the interfacial area available during emulsion formation and may facilitate the participation of surface‐active proteins and phospholipids at the oil–water interface (Ravera et al. 2021). The reduction in particle size may have contributed to the increased emulsifying activity and emulsion stability observed in Section 3.1.1 by improving particle dispersion and increasing the available interfacial area. Additionally, smaller particles also reduce gravitational separation velocity according to Stokes' law, thereby improving creaming stability (Chanamai and McClements 2000).

Similarly, Geng et al. (2021) showed that ultrasound treatment depolymerized egg yolk granules into smaller particles by disassembling lipoprotein–phosvitin networks, enhancing particle dispersion and emulsifying properties. More recently, Li et al. (2026) reported that ultrasound‐assisted pH‐shifting modified the organization of egg yolk granule–lecithin complexes and their interfacial behavior. Appropriate protein–phospholipid interactions promoted the formation of a viscoelastic interfacial layer and were associated with smaller emulsion droplets, a lower creaming index, and greater interfacial protein coverage. Although the treatment conditions differed from those used in the present study, these findings suggest that changes in the organization and dispersion of yolk components may contribute to improved interfacial behavior and emulsion stability following ultrasound‐related processing. The importance of egg yolk granule organization for emulsion stability has also been demonstrated in other yolk‐based emulsion systems. Sun et al. (2025) showed that egg yolk granules combined with chitosan formed colloidal particles capable of stabilizing low‐fat Pickering emulsion gels, with emulsion structure and stability strongly dependent on particle organization and oil‐phase fraction. Although this system differs from the ultrasound‐treated liquid egg yolk examined in the present study, these findings further indicate that the dispersed state and organization of egg yolk granules can play an important role in emulsion stability. Some studies observed that excessive ultrasound can promote recoalescence or protein aggregation, leading to an increase in particle size (Teng et al. 2020; Xie et al. 2020). However, the particle size of egg yolk continued to decrease during 20 min cylindrical focused ultrasound treatment in the present study, indicating that the applied cylindrical focused ultrasound conditions remained within an effective range for structural change of egg yolk rather than overprocessing.

Furthermore, to visually examine whether the reduction in egg yolk particle size was reflected in the droplet structure of the egg yolk emulsions, optical microscopy was performed to observe the emulsion droplets. For each treatment, three images of optical microscopic images were captured, and the most representative image was selected to illustrate the droplet morphology of the egg yolk emulsion (Figure 3B). The untreated egg yolk contained larger, polydisperse droplets with uneven spatial distribution. However, the egg yolk treated with cylindrical focused ultrasound for 5 min displayed fewer large droplets. Moreover, the egg yolk treated for 10 min showed smaller droplets with improved uniformity, and the egg yolk treated for 20 min presented the finest and most homogeneous droplet field, with very few visibly large droplets. The formation of smaller and more uniform droplets indicates improved emulsion formation and may be associated with the higher emulsifying activity and emulsion stability observed in the ultrasound‐treated egg yolk.

### 3.3 Surface Hydrophobicity

To evaluate cylindrical focused ultrasound induced changes in interfacial protein exposure of egg yolk, surface hydrophobicity of egg yolk was measured using ANS fluorescence across protein concentrations of 0.1–0.5 mg/mL. Fluorescence intensity increased linearly with concentration for all egg yolk (Figure 4). The slope (hydrophobicity index) was 569.40 for the untreated egg yolk and 605.22, 611.90, and 633.41 for the samples treated with cylindrical focused ultrasound for 5, 10, and 20 min, respectively. The ANS surface hydrophobicity of egg yolk was significantly increased by cylindrical focused ultrasound treatment (5, 10, and 20 min), but there was no significant difference among the treatment times.

**FIGURE 4:**
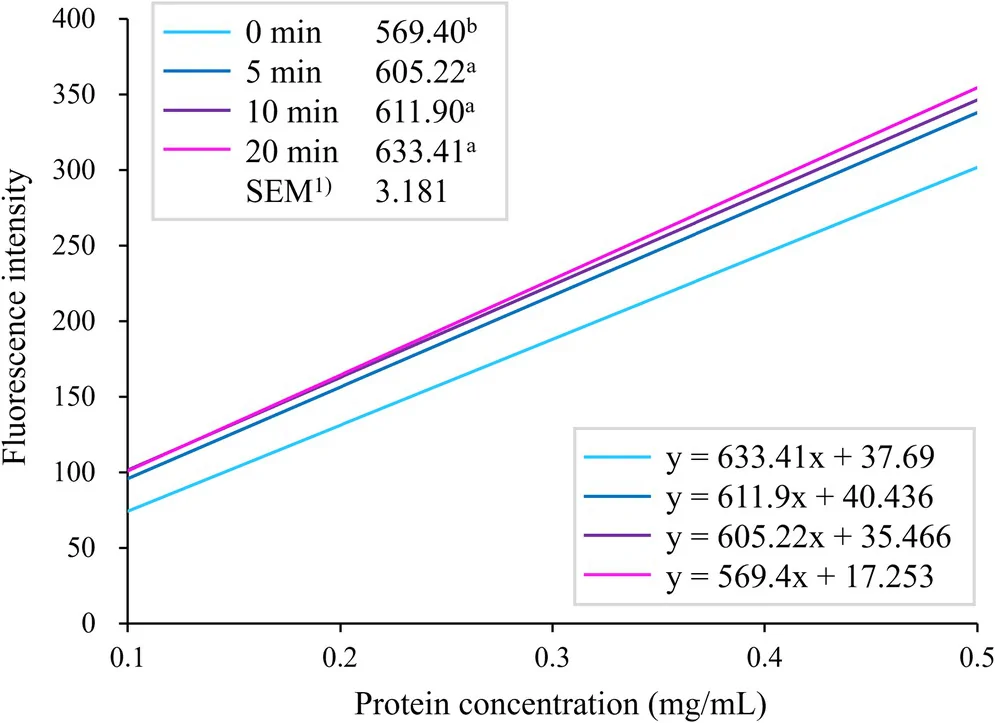
Effect of cylindrical focused ultrasound treatment time on the surface hydrophobicity of egg yolk. ^a,b^Values with different superscripts differ significantly (*p* < 0.05) among treatments. ^1^Standard error of the means (*n* = 12).

The increase in surface hydrophobicity may indicate greater accessibility of hydrophobic regions in egg yolk proteins or protein–lipid complexes following cylindrical focused ultrasound treatment (Ma et al. 2018). Ultrasound‐induced increases in surface hydrophobicity have previously been associated with conformational rearrangement and changes in the organization of protein‐based structures. Therefore, the increased surface hydrophobicity observed in the present study may reflect changes in the organization or exposure of hydrophobic regions within egg yolk components.

Chemical activity under the applied cylindrical focused ultrasound was evaluated using the iodide oxidation method (Figure 5). I_3_^−^ absorbance increased with cylindrical focused ultrasound treatment time, indicating increased chemical oxidative activity in the aqueous KI system. These results are consistent with the increased surface hydrophobicity observed in cylindrical focused ultrasound‐treated egg yolk. Therefore, chemical reactions may have contributed to the increased surface hydrophobicity. In general, proteins or protein–lipid complexes with higher surface hydrophobicity can adsorb more effectively at oil–water interfaces and form more cohesive interfacial films, thereby contributing to emulsion stabilization in Section 3.1 (Kallakas et al. 2024). Therefore, the accessibility of hydrophobic regions may facilitate interactions of surface‐active yolk components with the oil phase and contribute to the improved emulsifying properties observed in the present study. Consistent with this study, Ma et al. (2025) found that ultrasound treatment of egg yolk increased surface hydrophobicity, which correlated with higher oil exudation. However, further study using protein structure analysis is needed to confirm whether cylindrical focused ultrasound induces protein oxidation, weakens protein‐phospholipid interactions, or changes the composition of protein.

**FIGURE 5:**
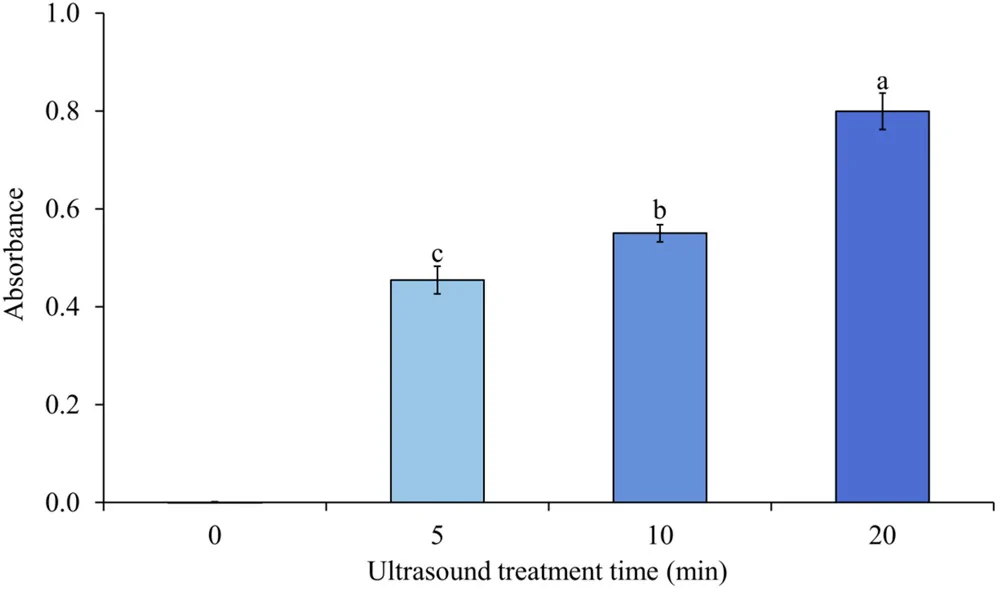
Determination of chemical effect by cylindrical focused ultrasound treatment time using iodide oxidation absorbance method. ^a–c^Values with different superscripts differ significantly (*p* < 0.05) among treatments.

### 3.4 TBARs Values

MDA, a secondary marker formed during the oxidation of polyunsaturated fatty acids, was quantified by the TBARs assay and expressed as mg MDA/L (Figure 6A). Compared to the untreated egg yolk, TBARs values of egg yolk treated with cylindrical focused ultrasound exhibited significantly higher (*p < 0.05*). The TBARs values of egg yolk treated with cylindrical focused ultrasound for 5 and 10 min were higher than those of the untreated egg yolk (*p < 0.05*), showing an increase of 0.08 mg MDA/L for both treatments, with no significant difference. The egg yolk treated with cylindrical focused ultrasound for 20 min resulted in the highest TBARs value (1.196 mg MDA/L), which was 0.43 mg MDA/L higher than that of the untreated egg yolk (0.767 mg MDA/L).

**FIGURE 6:**
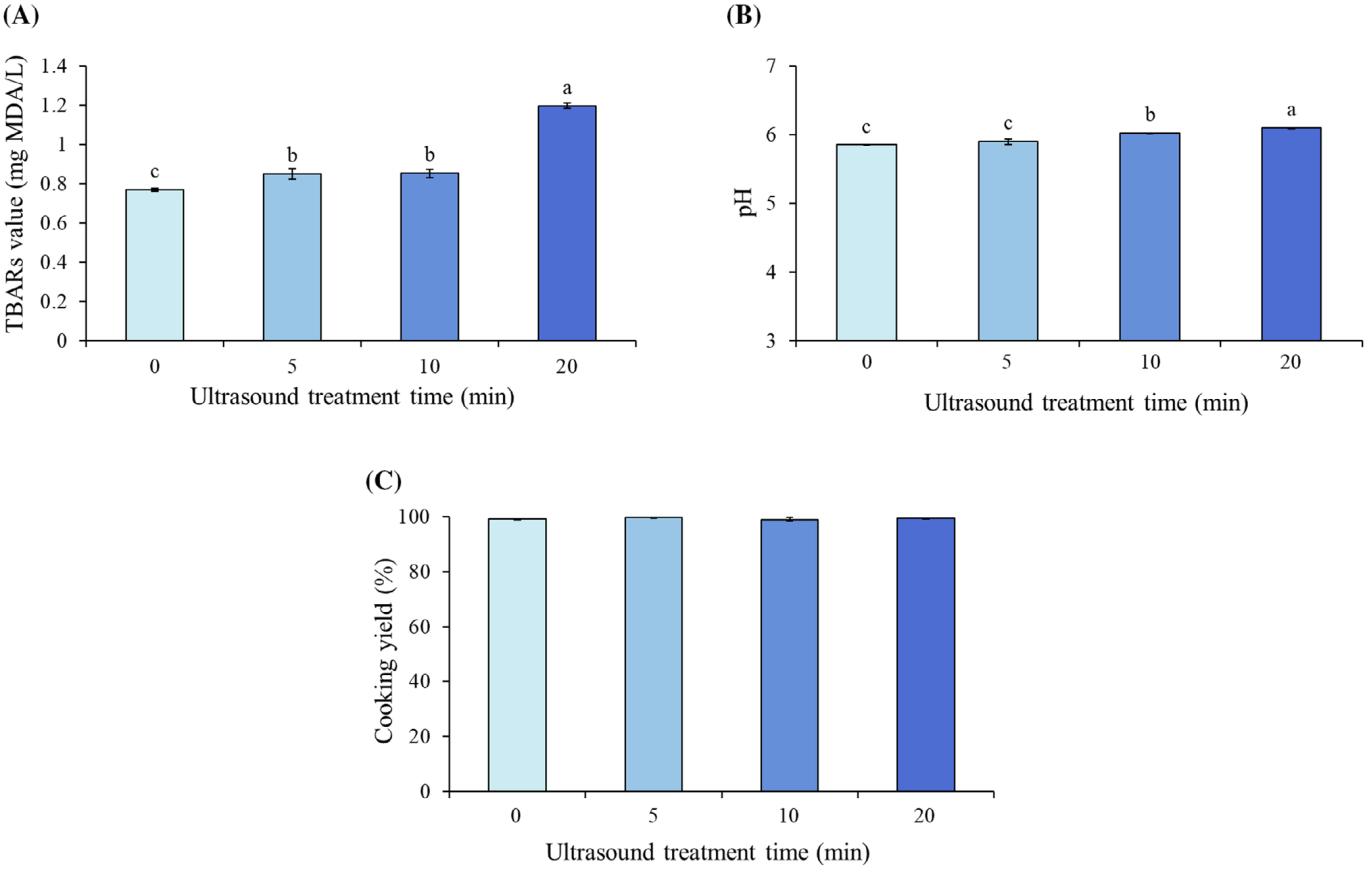
Effect of cylindrical focused ultrasound treatment time on the physicochemical properties of egg yolk. (A) TBARs value, (B) pH, (C) cooking yield. ^a–c^Values with different superscripts differ significantly (*p* < 0.05) among treatments.

During cylindrical focused ultrasound treatment, acoustic cavitation in egg yolk not only generates physical effects but also chemical effects. For example, cavitation can lead to a chemical effect that homolyzes water to produce reactive oxygen species (•OH, H_2_O_2_) (Rathnayake, Kim, et al. 2025). In the aqueous iodide oxidation assay, I_3_^−^ absorbance significantly increased with treatment time, indicating increased chemical oxidative activity under the applied cylindrical focused ultrasound (Figure 5). Although this assay does not directly demonstrate hydroxyl‐radical formation in the egg‐yolk matrix, the increased lipid oxidation observed after cylindrical focused ultrasound treatment may be associated with sonochemically induced oxidative reactions.

Similar increases in lipid oxidation after ultrasound treatment have been reported in egg yolk. Wang et al. (2021) applied ultrasonic‐assisted cooking to salted egg yolks using a KQ5200DE ultrasonic cleaner at 160 W and 80°C for 40 min. They reported that TBARs values in ultrasound‐assisted cooked salted egg yolks significantly increased compared with conventionally cooked salted egg yolk. They suggested that lipid oxidation was intensified by ultrasound because acoustic cavitation can generate localized high temperature, high pressure, and free radicals. This previous finding supports the present result that focused ultrasound treatment increased lipid oxidation in the lipid‐rich egg yolk matrix.

In general, foods are considered acceptable for consumption when their TBARs value remains below 3 mg MDA/L, while values in the range of approximately 7–8 mg MDA/L are often regarded as an upper tolerable limit (Rezaei et al. 2019). Therefore, the highest TBARs value observed in the present study (1.196 mg MDA/L) indicates an acceptable level for consumption. Nevertheless, the significant increase in TBARs indicates that prolonged cylindrical focused ultrasound treatment may compromise the oxidative stability of egg yolk, despite the relatively low absolute TBARs values observed immediately after treatment. However, because storage stability was not evaluated in the present study, the implications of the increased TBARs values for product quality and shelf life remain to be determined through further storage studies. Thus, the improvement in emulsifying functionality achieved by cylindrical focused ultrasound should be balanced against the increased susceptibility to lipid oxidation, particularly at prolonged treatment times.

### 3.5 pH

The pH values of egg yolk increased with longer cylindrical focused ultrasound time (Figure 6B). The pH values of egg yolk treated with cylindrical focused ultrasound for 10 and 20 min were higher than those of the untreated egg yolk and the egg yolk treated with cylindrical focused ultrasound for 5 min (*p* < 0.05), with the highest value at 20 min cylindrical focused ultrasound treatments.

Previous studies showed that pH changes in liquid egg after ultrasound treatment are generally small, but the change of egg yolk can vary depending on processing conditions and the egg matrix. For example, Yüceer (2019) reported that ultrasound treatment of liquid egg yolk at 100 and 250 W for 30 s decreased pH from 6.33 ± 0.03 as ultrasound power increased. However, Bi et al. (2020) observed that high‐power ultrasound treatment of liquid whole egg at 35°C–45°C and 605–968 W/cm^2^ for 5–35 min significantly increased pH. These previous findings indicate that the pH change in the egg induced by ultrasound is nonuniform and depends on treatment conditions.

In the present study, the slight increase in egg yolk pH may be associated with focused ultrasound‐induced dispersion or partial rearrangement of egg yolk components, which could alter the exposure of ionizable groups in the aqueous phase (Geng et al. 2021). However, all measured pH values remained within the typical range reported for fresh egg yolk, approximately 5.8–6.2 (Sharp and Powell 1931). Therefore, although cylindrical focused ultrasound treatment slightly increased the pH of liquid egg yolk, the change was limited and did not indicate substantial acidification or alkalization of the egg yolk system.

### 3.6 Color Values

The increase in CIE L*values of egg yolk treated with cylindrical focused ultrasound for 10 and 20 min was observed compared with untreated egg yolk, whereas no significant difference was observed at CIE a*, CIE b* values in all egg yolks (Table 1). ΔE, which means color difference with 0 min control, was calculated by this result. There was no significant difference between the egg yolk treated with cylindrical focused ultrasound for 5 and 10 min (*p* > 0.05), whereas that of egg yolk treated with cylindrical focused ultrasound for 20 min had significantly higher ΔE than egg yolk treated with cylindrical focused ultrasound for 5 min.

**TABLE 1:** Effect of cylindrical focused ultrasound treatment time on the color of egg yolk.

Ultrasound treatment time (min)	CIE L*	CIE a*	CIE b*	ΔE*
0	59.80^c^	14.03	80.60	0.00^c^
5	61.51^bc^	15.34	81.19	2.28^b^
10	62.26^ab^	15.57	78.70	4.04^ab^
20	63.59^a^	15.32	79.30	4.60^a^
SEM^1^	0.201	0.226	0.284	0.195

^a–c^Values with different letters within the same column differ significantly (*p* < 0.05).

^1^ Standard error of the means (*n* = 12).

As shown in Figure 3A, ultrasound‐induced cavitation reduces the particle size distribution of egg yolk. A finer dispersion of particles enhances light scattering within the egg yolk, where the CIE L* value of egg yolk treated with cylindrical focused ultrasound was increased, indicating a brightening of the yolk color (Fujii et al. 2024). In contrast to the changes in CIE L* values of egg yolk, the CIE a* and CIE b* values have no significant differences observed among the treatments. This suggests that cylindrical focused ultrasound treatment primarily affected the physical structure of egg yolk rather than the chemical composition of the chromophores responsible for color characteristics. Carotenoids are the primary chromophores responsible for the characteristic yellow‐orange color of egg yolk (Islam et al. 2017). In the CIE L*a*b* system, CIE a* and CIE b* mainly represent chromatic attributes associated with the spectral absorption of yolk carotenoids, whereas CIE L* is more sensitive to overall lightness and light scattering. Therefore, the unchanged CIE a* and CIE b* values of egg yolk suggest that natural pigments such as carotenoids were not substantially altered or degraded under the cylindrical focused ultrasound conditions used in this study (Itle and Kabelka 2009). Consistent with the present study, Yoshimasa et al. (2024) found that ultrasound of liquid egg yolk increased in CIE L* value with increasing ultrasound intensity and treatment time.

### 3.7 Cooking Yield

Cooking yield was additionally evaluated to determine whether cylindrical focused ultrasound‐induced structural modifications adversely affected the thermal water‐retention properties of egg yolk during heating. As shown in Figure 6C, the changes in cooking yield of egg yolk with or without cylindrical focused ultrasound treatment were not significantly different (*p* > 0.05). Cooking yield of egg yolk was higher than 99% in all of the treatments.

Cooking yield is commonly used as an indicator of the ability of the protein network to retain moisture during thermal processing (Zheng et al. 2024). Gelation of globular proteins on heating involves partial unfolding of the native globular structure followed by intermolecular aggregation (Clark et al. 2001). Previous studies have shown that ultrasound‐assisted thermal processing can adversely affect cooking yield, depending on treatment conditions and the food matrix. Wang et al. (2021) applied ultrasonic‐assisted cooking to salted egg yolks at 40 kHz and 160 W for 40 min at 80°C and reported that cooking loss significantly increased compared with conventionally cooked salted egg yolks. In contrast, no significant difference in cooking yield was observed between untreated and cylindrical focused ultrasound‐treated egg yolk in the present study (*p* > 0.05). The maintained cooking yield observed in the present study suggests the potential applicability of cylindrical focused ultrasound‐treated egg yolk in thermally processed emulsion‐based food systems. However, the underlying mechanisms by which ultrasound modulates cooking loss in different food matrices still require.

## 4 Conclusion

Cylindrical focused ultrasound improved the emulsifying activity, emulsion stability, and creaming stability of liquid egg yolk under the conditions evaluated in this study, with the 20 min treatment generally showing the greatest improvement. These functional changes were accompanied by reduced particle size and increased surface hydrophobicity, suggesting that changes in the dispersion and surface characteristics of egg yolk components may contribute to the improved emulsifying properties. However, TBARs values increased with treatment time, indicating that prolonged cylindrical focused ultrasound treatment may increase lipid oxidation. At the same time, cooking yield was not affected, and only limited changes in color were observed.

Overall, these results indicate that cylindrical focused ultrasound has potential for improving the emulsifying functionality of liquid egg yolk under the conditions evaluated in this study. However, the concurrent increase in lipid oxidation indicates a trade‐off between improved emulsifying functionality and oxidative stability with prolonged treatment. Therefore, optimization of treatment conditions should balance these functional benefits against oxidative changes. Further evaluation under storage and actual food‐system conditions is needed before broader practical application. In addition, scale‐up studies evaluating processing capacity and energy efficiency are required to determine the feasibility of cylindrical focused ultrasound for industrial application.

## Author Contributions

**Hyun Uk Cho:** writing – original draft, software, data curation, validation, conceptualization, methodology, investigation, formal analysis. **Chemin Nam:** writing – review and editing, data curation, validation, conceptualization, methodology. **Prabhathma Yasasvi Rathnayake:** conceptualization, data curation, formal analysis, methodology, validation. **So Eun Yeo:** investigation, methodology, data curation, validation. **Seonae Hwangbo:** conceptualization, methodology, resources. **Ki Ho Baek:** conceptualization, data curation, methodology, software, investigation, validation. **Hae In Yong:** conceptualization, methodology, supervision, project administration, writing – review and editing, resources, validation, data curation, formal analysis.

## Funding

This work was supported by the National Research Foundation of Korea (NRF) grant funded by the Korea government (MSIT) (Project No. RS‐2025‐24523920).

## Ethics Statement

The authors have nothing to report.

## Consent

Written informed consent was obtained from all study participants.

## Conflicts of Interest

The authors declare no conflicts of interest.

## Data Availability

The data that support the findings of this study are available from the corresponding author upon reasonable request.
